# Coexistence and correlates of forms of malnutrition among mothers and under-five child pairs in Tanzania

**DOI:** 10.1017/jns.2022.103

**Published:** 2022-11-22

**Authors:** Fina T. Faustini, Ali R. Mniachi, Amina S. Msengwa

**Affiliations:** Department of Statistics, College of Social Sciences, University of Dar es Salaam, P.O. BOX 35047, Dar es Salaam, Tanzania

**Keywords:** Double burden of malnutrition, Malnutrition, Overweight, Underweight, LBW, low birth weight, RRR, relative risk ratio, TDHS-MIS, Tanzania Demographic Health Survey and Malaria Indicator Survey, WHO, World Health Organization

## Abstract

Improving the health and well-being of mothers and children is a priority worldwide. The present study aimed to examine the coexistence and correlates of malnutrition among mothers and under-five child pairs using Tanzania Demographic Health Survey 2015–16 data. Height-for-age, height-for-weight and weight-for-age *Z*-scores were used to assess the nutritional status of children, while body mass index was used to assess the nutritional status of mothers. Correlates of forms of malnutrition were assessed using multinomial logistic regression. Among 8083 pairs, 40⋅9 % were normal, 30⋅3 % were underweight, 17⋅5 % overweight and 11⋅3 % had double burden of malnutrition. The risk of being underweight is highest among the pairs with; children aged 13–59 months (relative risk ratio (RRR) = 2⋅33) and children with small birth weight (RRR = 2⋅67). Overweight is highest among pairs with; mothers aged 35–49 (RRR = 3⋅36), mothers with secondary education and above (RRR = 1⋅85), fathers aged 35+ (RRR = 1⋅38), professional fathers (RRR = 4⋅10) and richer households (RRR = 2⋅06). The double burden of malnutrition is highest among pairs with; children with small birth weight (RRR = 2⋅76), from rural households (RRR = 1⋅24) and from richer households (RRR = 1⋅41). There is a coexistence of forms of malnutrition among mothers and under-five child pairs in Tanzania. The study recommends using multidimensional approaches such as double-duty action for nutrition to eradicate all forms of malnutrition.

## Introduction

For the past four decades, malnutrition has gained remarkable consideration in the health sector and governments worldwide. The considerations have stemmed from its impact. Although malnutrition has several consequences, researchers have pointed out the most severe outcomes: poor fetal growth, low birth weight (LBW), short and long-term maternal and infant morbidity, and mortality^(1,2)^.

Over the years, most studies on nutritional status have focused on individual-level, either children, adolescents, women or adults. The nutritional status of women of reproductive age has a significant impact on the nutritional status of children since there is a link between maternal and child nutrition. Maternal malnutrition is associated with low birth weight, stillbirth and maternal mortality^(3)^. A malnourished mother is more likely to give birth to an undernourished child who will grow up to be undernourished and hence a vicious cycle of malnutrition^(4)^. Therefore, it is crucial to study the nutritional status of mother–child pairs and the associated factors to address the coexistence of malnutrition among mother–child pairs.

Many African countries have managed to reduce malnutrition in children under five; however, the situation is still worse. Recent statistics indicate that more than 59 million children under-five years are stunted, 10 million are fighting to reduce their weight and 69 million suffer from wasting. Despite the decrease in the prevalence of stunting globally, the number of stunted children in Africa has increased due to population growth^(5)^.

Tanzania is not excluded from the countries with a high level of malnutrition. Various reports, including that of TDHS-MIS presented in 2016, show that 34 % of under 5 years old children are stunted, 5 % are wasted, 14 % are underweight and 4 % are overweight. The same document indicates that stunting is higher in Tanzania (35 %) than in Zanzibar (24 %). The prevalence of stunting among under 5 years old children is most elevated in Rukwa (56 %), Njombe (49 %) and Ruvuma (44 %). The report also indicates that 10 % of women of reproductive age are underweight while 28 % are overweight. Hence, the report shows how malnutrition affects children and women of reproductive age.

There are studies on malnutrition among under 5 years old across countries^(6–9)^. In addition, some studies have reported an emerging health problem of the coexistence of forms of malnutrition among mother–child pairs^(10)^. However, there is limited research on the nutritional status of mothers and under-five child pairs living in the same household in Tanzania. The availability of data on the nutritional status of under 5 years old children and women of reproductive age provides an opportunity to use statistical techniques to examine forms of malnutrition among mother–child pairs jointly.

Therefore, the present study aims to investigate forms of malnutrition among mothers and under-five child pairs in Tanzania. The first objective was to examine the prevalence and coexistence of forms of malnutrition among mother–child pairs. The second objective was to assess correlates and risk factors of forms of malnutrition among mother–child pairs. The study's goal was to add to our understanding of forms of malnutrition among mother–child pairs in Tanzania. Hence, emphasise double-duty action plans for nutrition to end malnutrition and all its forms simultaneously.

## Materials and methods

### Data

This study used a cross-sectional research design using the Tanzania Demographic Health Survey and Malaria Indicator Survey (TDHS-MIS) 2015–16 data. The survey is the sixth series of national health surveys conducted in Tanzania every 5 years. The survey provides updated information on population, marriage and sexual activity, fertility, family planning, infant and child mortality, maternal and health care, child health, nutrition of children and women, malaria, adult and maternal mortality, women's empowerment, female genital cutting and domestic violence. The dataset has nationally representative information on the nutritional status of 13 376 households. The information includes anthropometric measures, iodine and anaemia tests for under 5 years old children and women aged 15–49 years at the time of the survey.

Data collection was sponsored by the Agency for International Development and coordinated by Macro International. The data are nationally representative and collected in collaboration with the Tanzanian government through the National Bureau of Statistics using population sampling frames. The sampling frame used the Tanzania Population and Housing Census conducted in 2012, consisting of a list of enumeration areas covering Tanzania.

**Fig. 1.**
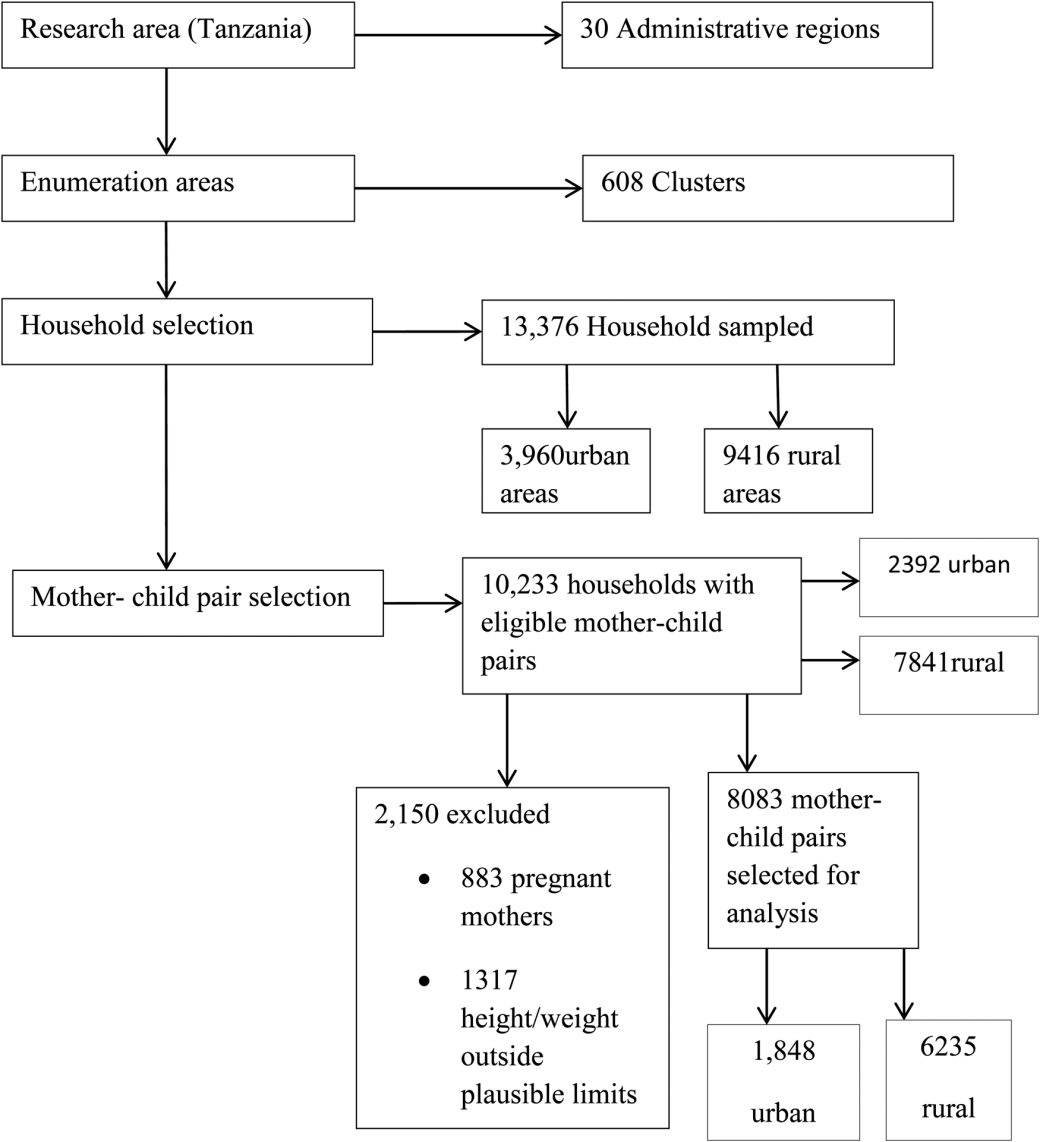
A diagram showing a sample of 8083 mother–child pairs extracted from the Tanzania Demographic Health Survey (TDHS-MIS 2015–16).

With the design, the survey selected 13 376 households. From the selected households, 10 233 were used to assess the nutritional status of under-five children and their mothers. The present study has screened data for mothers and under-five children with weight and height measurements available. In addition, the study excluded pregnant mothers and the pair with measurements beyond reasonable limits. Therefore, a study used a sample of 8083 mother–child pairs extracted from TDHS-MIS 2015–16 data, as shown in Fig. 1.

### Variables

#### Dependent variable

The dependent variable is the nutritional status of mothers and under-five child pairs as:1
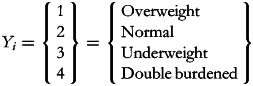


Body mass index (BMI) was used as an indicator of the nutritional status of mothers. In contrast, height-for-age *Z*-score (HAZ), weight-for-height *Z*-score (WHZ) and weight-for-age *Z*-score (WAZ) indicators were used to assess the nutritional status of the under-five children. A child is considered undernourished if stunted, wasted or underweight, and is normal when it is neither stunted, wasted or underweight. In addition, the child is stunted, wasted or underweight if HAZ, WHZ and WAZ is −2 sd below the median of the respective index of the referenced population.

The BMI categorises a mother's weight as normal, underweight or overweight according to WHO references.

#### Independent variables

The independent variables include the following characteristics: sex of a child (male, female), age of a child (0–2, 3–12, 13–59 months), birth weight of a child (low if weight < 2500 g, normal and above if weight ≥2500 g), ever experienced fever/diarrhoea 2 weeks prior the survey (Yes, No). A child is considered to have diarrhoea if they had at least three loose or liquid stools per day, breast-feeding status (currently breast-feeding, not breast-feeding and ≤2 years of age and not breast-feeding and >2 years of age), ever had the vaccine? (Yes, No) and age of a mother (<25, 25–29, 30–34 and 35–49). Also, the highest education level of mother and father (no education, primary, secondary and above), marital status (single, married and divorced/separated/widowed) and occupation status of a mother and father (unemployment, agriculture, services and manual and professional). The study also included alcohol consumption habit of a mother (yes, no), age of a father (below 35 years and 35 years and above), number of under-five children in the household (1–2 and 3 and above) and family size (less than 5, 5 and above). In addition to that, sex of household head (male, female); type of place of residence (rural, urban); source of drinking water (improved, unimproved); type of toilet facility (improved, unimproved) and household wealth (poor, middle and richer).

### Data analysis

The study utilised both descriptive and inferential statistical methods to analyse the data. The analysis of the nutritional status of mothers and under-five child pairs was performed in the STATA version 14 statistical package. Bivariate analysis was used to determine the explanatory variables that are significantly associated with the nutritional status of mothers and under-five child pairs. Statistically significant variables at *α* = 5 % were used in a multinomial model to analyse the correlates and the risk factors of each form of malnutrition among mother–child pairs. In a multinomial model, normal nutritional status is used as a reference category for overweight, underweight and double burden categories.

## Results

**Fig. 2.**
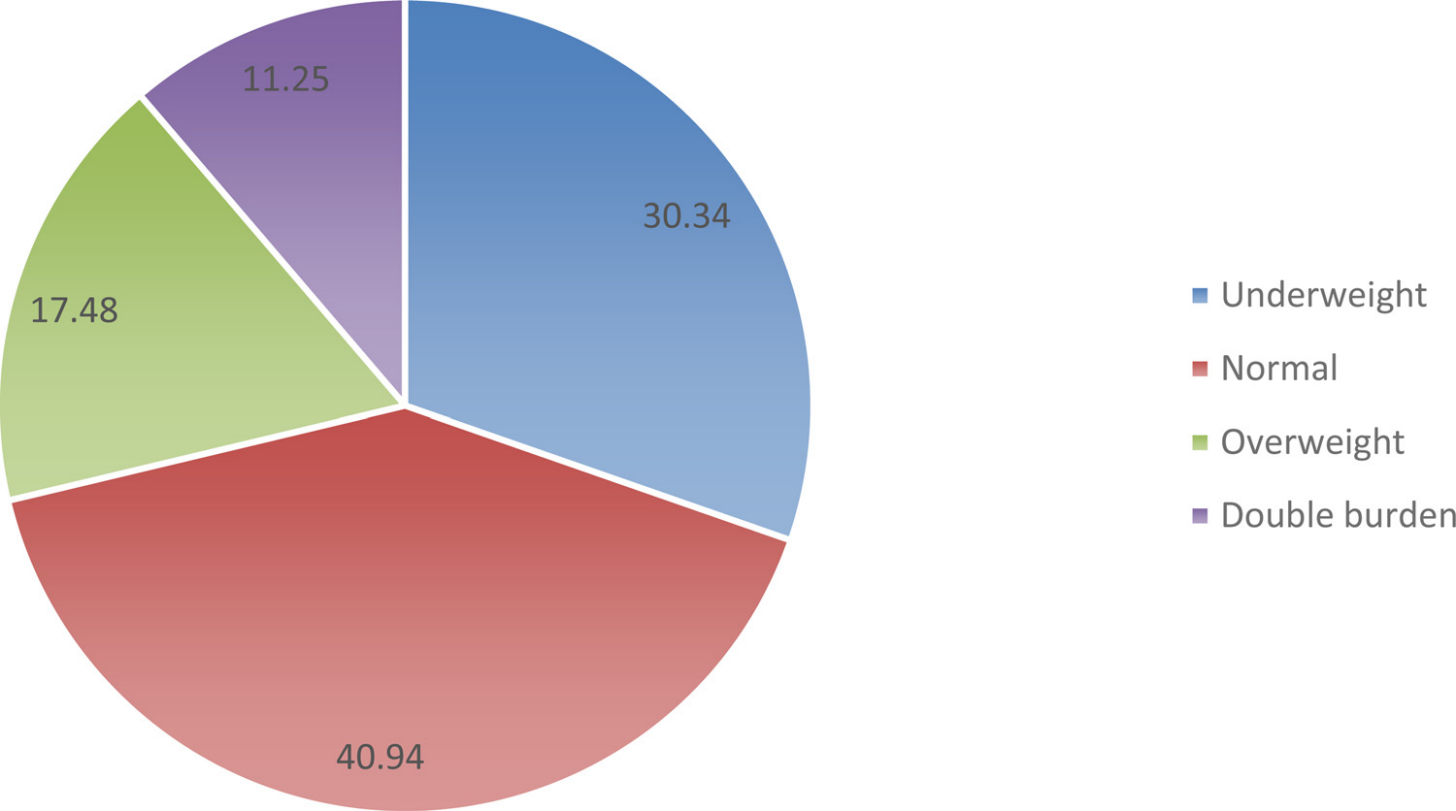
Prevalence of forms of malnutrition among mothers and under-five child pairs in Tanzania. Findings from Tanzania Demographic Health Survey show that 41 % of the pairs are normal, 30 % underweight, 18 % overweight and 11 % have double burden of malnutrition.

The study shows the coexistence of forms of malnutrition among mothers and under-five children living in the same families. The results in Fig. 2 show that 40⋅94 % of the pairs are normal, while 59⋅06 pairs are malnourished. Among the malnourished pairs, 11⋅25, 30⋅34 and 17⋅48 % have double burden, underweight and overweight nutritional status, respectively. In addition, the bivariate analysis showed that: the age and sex of a child; birth weight; breast-feeding status; age and education level of a mother; age and education level of a father; marital status; occupation status of a mother and father; the number of under-five children in the household; place of residence; source of drinking water; toilet facility and household wealth are associated with nutritional status of mother–child pairs.

### Risk of underweight among mothers and under-five child pairs

The multinomial logistic regression of underweight among mothers and under-five child pairs is shown in Table 1, reading from columns headed RRR. It is indicated that mothers and under-five child pairs with female children are 1⋅13 times less likely to be underweight than those with male children. The risk of underweight pairs is highest among the pairs with children aged 13–59 months (2⋅33 times more likely) than those aged 0–2 months. The pairs with children born with small weight are 2⋅67 times more likely to be underweight than those born with average or larger weights. Mothers and under-five child pairs with children who have received at least one vaccination are 1⋅24 times more likely to be underweight than pairs with children who have never received any vaccine. The pairs with mothers aged 25–29 years are 1⋅25 less likely to be underweight than the pairs with mothers aged <25 years. It is revealed that the mothers’ professional/managerial occupation status reduces the risk of underweight pairs whereby the pairs are 2⋅08 times less likely to be underweight than the pairs with unemployed mothers. The pairs with fathers aged 35+ are 1⋅01 times less likely to be underweight than the pairs with fathers aged <35 years. Furthermore, the pairs with fathers with secondary education and above are 1⋅27 times less likely to be underweight than the pairs with non-educated fathers. It was also observed that mothers and under-five child pairs from richer households are 1⋅38 times less likely to underweight than those from poor households.

**Table 1.** Bivariate analysis and multinomial logistic regression of malnutrition among mothers and under-five child pairs

Forms of malnutrition								
Variable	Underweight		Overweight		Double burden			
	Prev. (%)	RRR (95 % CI), *P*-value	Prev. (%)	RRR (95 % CI), *P*-value	Prev. (%)	RRR (95 % CI), *P*-value	Total	*χ*^2^ *P*-value
Child's characteristics								
Sex								
Male	31⋅7	1⋅0	16⋅5	1⋅0	11⋅6	1⋅0	4066	0⋅008*
Female	29	0⋅883 (0⋅785–0⋅992), 0⋅037*	18⋅5	1⋅107 (0⋅959–1⋅277), 0⋅165	10⋅9	0⋅961 (0⋅817–1⋅131), 0⋅633	4017	
Age								
0–2	20⋅5	1⋅0	19⋅9	1⋅0	6⋅8	1⋅0	517	0⋅000*
3–12	23⋅4	1⋅113 (0⋅835–1⋅484), 0⋅464	16⋅7	0⋅8 (0⋅587–1⋅09), 0⋅157	8⋅6	1⋅178 (0⋅761–1⋅822), 0⋅463	1622	
13–59	33⋅1	2⋅333 (1⋅749–3⋅113), 0⋅000*	17⋅5	0⋅686 (0⋅496–0⋅947), 0⋅022*	12⋅4	1⋅911 (1⋅237–2⋅952), 0⋅003*	5944	
Birth weight								
Average or larger	29⋅9	1⋅0	17⋅7	1⋅0	10⋅9	1⋅0	7758	0⋅000*
Small	41⋅2	2⋅672 (1⋅921–3⋅715), 0⋅000*	12⋅3	1⋅066 (0⋅687–1⋅653), 0⋅777	20⋅6	2⋅755 (1⋅855–4⋅091), 0⋅000*	325	
Had fever 2 weeks before the survey								
No	30⋅1	NA	17⋅7	NA	11	NA	6645	0⋅264
Yes	31⋅5	NA	16⋅4	NA	12⋅2	NA	1438	
Had diarrhoea 2 weeks before the survey								
No	30⋅3	NA	17⋅7	NA	11	NA	7081	0⋅275
Yes, last 2 weeks	30⋅3	NA	16	NA	12⋅8	NA	1002	
Breast-feeding status								
Currently breast-feeding	27⋅6	1⋅0	16⋅3	1⋅0	9⋅29	1⋅0	3423	0⋅005*
Not currently breast-feeding and ≤2 years	32⋅7	1⋅162 (0⋅905–1⋅492), 0⋅24	16⋅3	1⋅228 (0⋅896–1⋅683), 0⋅202	15⋅6	1⋅658 (1⋅204–2⋅284), 0⋅002*	627	
Not currently breast-feeding and >2 years	32⋅3	1⋅059 (0⋅877–1⋅279), 0⋅549	18⋅7	1⋅38 (1⋅079–1⋅766), 0⋅010*	12⋅2	1⋅227 (0⋅941–1⋅6), 0⋅131	4033	
Ever received vaccination?								
No	30⋅6	1⋅0	18⋅8	1⋅0	11⋅5	1⋅0	3057	0⋅018*
Yes	30⋅2	1⋅211 (1⋅035–1⋅418), 0⋅017*	16⋅7	1⋅045 (0⋅857–1⋅273), 0⋅664	11. 1	1⋅203 (0⋅966–1⋅499), 0⋅099*	5026	
Mother's characteristics								
Age								
<25	34⋅5	1⋅0	9⋅1	1⋅0	9⋅5	1⋅0	2338	0⋅000*
25–29	29	0⋅803 (0⋅681–0⋅947), 0⋅009*	18⋅2	1⋅944 (1⋅54–2⋅454), 0⋅000*	9	0⋅908 (0⋅704–1⋅171), 0⋅458	1959	
30–34	27⋅9	0⋅95 (0⋅777–1⋅163), 0⋅621	22⋅3	2⋅604 (1⋅994–3⋅4), 0⋅000*	12⋅2	1⋅362 (1⋅019–1⋅821), 0⋅037*	1576	
35–49	28⋅8	0⋅971 (0⋅786–1⋅198), 0⋅782	22⋅3	3⋅36 (2⋅545–4⋅436), 0⋅000*	14⋅3	1⋅802 (1⋅336–2⋅43), 0⋅000*	2210	
Highest educational level								
No education	35	1⋅0	11⋅1	1⋅0	11⋅2	1⋅0	1744	0⋅000*
Primary	31⋅6	0⋅997 (0⋅862–1⋅154), 0⋅968	15⋅4	1⋅228 (0⋅997–1⋅512), 0⋅053*	11	0⋅924 (0⋅748–1⋅142), 0⋅466	4840	
Secondary+	20⋅8	1⋅056 (0⋅83–1⋅344), 0⋅658	31⋅8	1⋅853 (1⋅408–2⋅439), 0⋅000*	12⋅2	0⋅995 (0⋅728–1⋅359), 0⋅973	1499	
Marital status								
Single	33⋅9	NA	12	NA	9⋅8	NA	410	0⋅000*
Married	29⋅7	NA	18⋅3	NA	11⋅4	NA	6829	
Divorced/separated/widowed	33⋅9	NA	13⋅7	NA	11	NA	844	
Occupation status								
Unemployed	29⋅2	1⋅0	19⋅7	1⋅0	12	1⋅0	1392	
Agriculture	35⋅3	0⋅803 (0⋅673–0⋅957), 0⋅014*	10⋅9	0⋅73 (0⋅585–0⋅911), 0⋅005*	9⋅7	0⋅729 (0⋅57–0⋅931), 0⋅011*	4553	0⋅000*
Services and manual	21⋅8	0⋅79 (0⋅644–0⋅97), 0⋅025*	28⋅4	1⋅206 (0⋅976–1⋅491), 0⋅083*	14⋅71	1⋅146 (0⋅891–1⋅474), 0⋅287	1922	
Professional	9⋅3	0⋅481 (0⋅27–0⋅856), 0⋅013*	45⋅1	1⋅091 (0⋅73–1⋅633), 0⋅67	13⋅3	0⋅8 (0⋅455–1⋅405), 0⋅437	226	
Ever consume alcoholic drink								
No	30⋅1	NA	17⋅5	NA	11⋅2	NA	7007	0⋅425
Yes	32⋅1	NA	17⋅4	NA	11⋅7	NA	1076	
Father's characteristics								
Age								
Below 35	32	1⋅0	13⋅2	1⋅0	9⋅3	1⋅0	2950	0⋅000*
35+	27⋅9	0⋅99 (0⋅843–1⋅163), 0⋅903	22⋅1	1⋅376 (1⋅127–1⋅68), 0⋅002*	12⋅9	1⋅171 (0⋅927–1⋅48), 0⋅185	3879	
Education level								
No education	35⋅9	1⋅0	11⋅6	1⋅0	10⋅8	1⋅0	1016	0⋅000*
Primary	31⋅5	0⋅906 (0⋅767–1⋅071), 0⋅248	15⋅7	1⋅006 (0⋅789–1⋅282), 0⋅964	10⋅9	0⋅961 (0⋅748–1⋅233), 0⋅752	4460	
Secondary+	19	0⋅789 (0⋅618–1⋅008), 0⋅058*	31⋅9	1⋅232 (0⋅914–1⋅66), 0⋅171	13⋅2	1⋅098 (0⋅79–1⋅525), 0⋅578	1353	
Occupation status								
Unemployed	31⋅9	1⋅0	5⋅8	1⋅0	13	1⋅0	69	0⋅000*
Agriculture	33⋅9	1⋅253 (0⋅721–2⋅177), 0⋅424	12⋅7	3⋅883 (1⋅327–11⋅362), 0⋅013*	10⋅6	1⋅217 (0⋅57–2⋅597), 0⋅612	4043	
Services and manual	24⋅8	1⋅289 (0⋅735–2⋅263), 0⋅376	25⋅3	3⋅771 (1⋅287–11⋅047), 0⋅015*	12⋅6	1⋅151 (0⋅534–2⋅481), 0⋅72	2367	
Professional	14⋅3	0⋅913 (0⋅475–1⋅751), 0⋅783	37⋅4	4⋅099 (1⋅358–12⋅372), 0⋅012*	11⋅4	0⋅976 (0⋅418–2⋅279), 0⋅955	350	
Household characteristics								
Number of under-five children in a household								
1–2	29	1⋅0	19⋅7	1⋅0	11⋅7	1⋅0	5911	0⋅000*
3+	33⋅9	0⋅991 (0⋅868–1⋅132), 0⋅895	11⋅4	0⋅769 (0⋅642–0⋅921), 0⋅004*	10	0⋅91 (0⋅749–1⋅105), 0⋅341	2172	
Family size								
Less than 5	29⋅2	NA	17⋅9	NA	10⋅8	NA	1658	0⋅535
5 and above	30⋅6	NA	17⋅4	NA	11⋅4	NA	6425	
Sex of household head								
Male	30⋅1	NA	17⋅8	NA	11⋅1	NA	6781	0⋅182
Female	31⋅7	NA	15⋅7	NA	12⋅1	NA	1302	
Type of place of residence								
Urban	20	1⋅0	30⋅5	1⋅0	12⋅2	1⋅0	1848	0⋅000*
Rural	33⋅4	1⋅094 (0⋅9–1⋅33), 0⋅366	13⋅6	0⋅821 (0⋅679–0⋅992), 0⋅041*	11	1⋅243 (0⋅981–1⋅574), 0⋅072*	6235	
Source of drinking water								
Improved source	26⋅8	1⋅0	21⋅3	1⋅0	12⋅6	1⋅0	4490	0⋅000*
Unimproved source	34⋅7	1⋅061 (0⋅934–1⋅205), 0⋅363	12⋅7	1⋅056 (0⋅895–1⋅247), 0⋅518	9⋅6	0⋅906 (0⋅754–1⋅089), 0⋅294	3593	
Type of toilet facility								
Improved source	18⋅1	1⋅0	33⋅1	1⋅0	14⋅5	1⋅0	2227	0⋅000*
Unimproved source	35	1⋅178 (0⋅949–1⋅464), 0⋅137	11⋅5	0⋅726 (0⋅587–0⋅898), 0⋅003*	10	0⋅841 (0⋅649–1⋅089), 0⋅188	5856	
Household wealth								
Poor	38	1⋅0	8⋅2	1⋅0	9⋅1	1⋅0	3514	0⋅000*
Middle	34⋅3	0⋅933 (0⋅798–1⋅09), 0⋅382	12⋅8	1⋅247 (0⋅993–1⋅564), 0⋅057*	10⋅7	1⋅065 (0⋅841–1⋅348), 0⋅601	1567	
Richer	19⋅3	0⋅724 (0⋅582–0⋅9), 0⋅004*	30⋅8	2⋅058 (1⋅6–2⋅647), 0⋅000*	14	1⋅409 (1⋅062–1⋅87), 0⋅017*	3002	
Constant		0⋅337 (0⋅169–0⋅674), 0⋅002*		0⋅039 (0⋅012–0⋅126), 0⋅000*		0⋅085 (0⋅033–0⋅224), 0⋅000*		
Mean dependent var		2⋅113		sd dependent var		0⋅96		
Pseudo R-squared		0⋅08		Number of observation		6829		
*χ*^2^		1398⋅646		Prob > *χ*^2^		0⋅00		
Akaike crit. (AIC)		16 308⋅308		Bayesian crit. (BIC)		16 882		

Prev. (%), prevalence of form of malnutrition; RRR, relative risk ratio; NA, not applicable.

* *P* < 0⋅05.

### Risk of overweight among mothers and under-five child pairs

It is observed in Table 1 that pairs with children aged 13–59 months are 1⋅46 times less likely to be overweight than pairs with 0–2 months children. The pairs with children who are not currently breast-feeding and are more than 2 years are 1⋅38 times more likely to be overweight than those who are currently breast-feeding. It was further revealed that the pairs with mothers aged 25 years and above have the highest risk of being overweight than the pairs with mothers aged less than 25 years. The pairs with mothers aged 35–49 are 3⋅36 times more likely to be overweight than those with mothers aged 25 and below. Moreover, pairs with mothers with primary and secondary education and above are 1⋅23 and 1⋅85 times more likely to be overweight than the pairs with mothers with no education. The results show that the pairs with services and manual work mothers are 1⋅1 times more likely to be overweight than the pairs with unemployed mothers. Contrary to that, the pairs with agricultural employed mothers are 1⋅37 times less likely to be overweight than the pairs with unemployed mothers.

The results show that the pairs with fathers aged 35 years and above are 1⋅38 times more likely to be overweight than those with younger fathers. It was further observed that the pairs with working fathers are more likely to be overweight than those with unemployed fathers. The highest risk is for those with professionally employed fathers (4⋅10 times more likely). The results also reveal that the households with more than three under-five children, from rural areas and with unimproved toilets facility are less likely to be overweight. Contrary to that, non-poor families have the highest risk of overweight among mothers and under-five child pairs as shown in Table 1.

### Risk of double burden of malnutrition among mothers and under-five child pairs

Table 1 reveals that the pairs with children born with weight below average are 2⋅76 times more likely to have a double burden of malnutrition than those born with average or larger weights. It was also observed that pairs with children who are not breast-fed and are at most 2 years have the highest risk (1⋅16 times more likely) of the double burden of malnutrition than those who are currently breast-feeding. Moreover, the pairs with children who received vaccination are 1⋅20 times more likely to have the double burden of malnutrition than those with children who never received the vaccination. The pairs with mothers aged 30 years and above are at the highest risk of the double burden of malnutrition than the pairs with younger mothers. Households from rural areas are 1⋅24 times more likely to have the double burden of malnutrition than households from urban areas. The results also showed that mothers and under-five child pairs from more affluent families are 1⋅41 times more likely to have the double burden of malnutrition than those from middle and poor households.

## Discussion

The present study assessed the coexistence of forms of malnutrition among mothers and under-five children living in the same households. It was observed that 11⋅25 % of the households have both a child and a malnourished mother. The prevalence is lower compared with that of a study conducted in Indonesia^(11)^ but higher than the prevalence observed by a study conducted in India^(12)^.

Results show that pairs with female children have the lowest risk of underweight than those with male children. This finding is different from findings on sex differences in nutritional status in Rwanda^(13)^, Senegal^(14)^ and Kenya^(15)^. However, it is consistent with a study in Gaza Strip^(16)^, Pakistan^(17)^ and Sub-Saharan Africa^(18)^. In addition, studies found that differences in this is due to their biological differences in morbidity in their early stages of life^(18,19)^. This may also be the same reason why a pair with female children have an increased risk of being underweight.

Multinomial logistic regression shows that pairs with mothers having secondary education and above are more likely to be overweight than those with no education. Also, those with fathers with secondary education are less likely to be underweight. Studies have found that being overweight is associated imbalance between calories intake and calories expended^(20)^. A high-income environment is associated with higher calories intake, and a higher education level is related to a high-income environment. This may also apply to our study that the pairs with mothers/fathers with secondary education and above are associated with a higher intake of calories and hence increased risk of overweight. However, the findings of the present study are contrary to the findings of the study conducted in Uganda^(20)^ and found no relation between malnutrition and parental education.

It is further observed that pairs from poor households have a higher risk of being underweight while pairs from wealthier families have the highest chance of being overweight and double burdened pairs. Similarly, studies in South Africa and Kenya found that mothers and children from wealthier wealth quintiles are at the highest risk of the double burden of malnutrition^(21,22)^ compared with those from poor wealth quintiles. Contrary, a study in Sri Lanka found no significant association between income and the double burden of malnutrition^(23)^. Wealthy households may be associated with reduced risks of being underweight because they have enough resources to address the nutritional needs of their members.

Findings show that having an employed mother is associated with a reduced risk of being underweight among the pairs. Furthermore, a pair with a mother with professional/managerial work is most likely associated with a reduced risk of underweight than those with unemployed mothers. These findings are similar to the study conducted in Ethiopia^(24)^, which reports that stunting, wasting and underweight are primarily observed in children with unemployed mothers. This may be because employment increases economic gain and positively impacts dietary intake and anthropometry of the pairs. The pairs with employed parent(s) might have adequate access to good nutrition as compared with the pairs with unemployed parent(s).

Furthermore, a pair with mothers working in agricultural activities are associated with a reduced risk of being overweight. This may result from physical exercises involved in agricultural activities as the job is labour-intensive. Therefore, farming activities can help burn calories and prevent overweight. Also, having a father with professional work is associated with an increased risk of overweight among the pairs. This further indicates that malnutrition affects all households regardless of the parent's employment status.

It is observed that being from a rural residence reduces the risk of being overweight while it increases the risk of the double burden of malnutrition among the pairs. This shows that both rural and urban areas are at risk of malnutrition among the pairs. Rural areas are affected by being underweight and double burden of malnutrition, while urban areas are affected by overweight. Previous studies on thirty-five developing countries^(25)^ also found that rural residency is associated with poor nutritional status. This may be because the rural residence is related to the low standard of living^(26)^, resulting in poor nutritional status.

## Conclusion

Sustainable Development Goal 2 emphasises that countries and stakeholders should work together to end hunger and all forms of malnutrition. Each socio-economic status is affected by different forms of malnutrition. Lower social economic is affected by being underweight, while higher socio-economic status is affected by the overweight and double burden of malnutrition. Hence, strategies to eradicate malnutrition in Tanzania should consider all forms of malnutrition to avoid the future increase in the prevalence of overweight and double burden of malnutrition in households. Households from higher socio-economic status should not be ignored in strategies to eradicate malnutrition. Emphasis should be placed on double-duty action plans. Strategies to eliminate all forms of malnutrition, such as providing education on healthy eating, should be provided to all households to improve the health status of all family members.

## References

[1] Black RE, Victora CG, Walker SP, (2013) Maternal and child undernutrition and overweight in low-income and middle-income countries. Lancet 382, 427–451. 23746772 10.1016/S0140-6736(13)60937-X

[2] Jones G, Steketee RW, Black RE, (2003) How many child deaths can we prevent this year? Lancet 362, 65–71. 12853204 10.1016/S0140-6736(03)13811-1

[3] Venkatachalam PS (1962) Maternal nutritional status and its effect on the newborn. Bull World Health Organ 26, 193–201. 13925322 PMC2555677

[4] Genebo T, Girma W, Haider J, (1999) The association of children's nutritional status to maternal education in Zigbaboto, Guragie Zone, Ethiopia. Ethiop J Health Dev 13, 55–61.

[5] Fanzo J, Hawkes C, Udomkesmalee E, (2019) 2018 Global Nutrition Report. London, UK: Global Nutrition Report.

[6] Aheto JMK, Keegan TJ, Taylor BM, (2015) Childhood malnutrition and its determinants among under-five children in Ghana. Paediatr Perinat Epidemiol 29, 552–561. 26332093 10.1111/ppe.12222

[7] Kandala NB, Madungu TP, Emina JB, (2011) Malnutrition among children under the age of five in the Democratic Republic of Congo (DRC): does geographic location matter? BMC Public Health 11, 261. 21518428 10.1186/1471-2458-11-261 PMC3111378

[8] Kazembe LN & Namangale JJ (2007) A Bayesian multinomial model to analyse spatial patterns of childhood co-morbidity in Malawi. European J Epidemiol 22, 545–556. 17565446 10.1007/s10654-007-9145-y

[9] Wand H, Lote N, Semos I, (2012) Investigating the spatial variations of high prevalences of severe malnutrition among children in Papua New Guinea: results from geoadditive models. BMC Res Notes 5, 228. 10.1186/1756-0500-5-228 PMC3814590 22574768

[10] Valente A, Borges A, Dias C, (2017) Relationship between the mothers’ nutritional status with that of a child population from São Tomé Principe, “Africa”. Rev Bras Saúde Materno Infant 17, 327–335.

[11] Rachmah Q, Mahmudiono T & Loh SP (2021) Predictor of obese mothers and stunted children in the same roof: a population-based study in the urban poor setting Indonesia. Front Nutr. Available from: https://www.frontiersin.org/articles/10.3389/fnut.2021.710588/full. 10.3389/fnut.2021.710588 PMC8687438 34938755

[12] Patel R, Srivastava S, Kumar P, (2020) Factors associated with double burden of malnutrition among mother-child pairs in India: a study based on National Family Health Survey 2015–16. Child Youth Serv Rev 116, 105256. 10.1186/s12889-021-10411-w PMC7901069 33622303

[13] Condo JU, Gage A, Mock N, (2015) Sex differences in nutritional status of HIV-exposed children in Rwanda: a longitudinal study. Trop Med Int Health 20, 17–23. 25345559 10.1111/tmi.12406

[14] Bork KA & Diallo A (2017) Boys are more stunted than girls from early infancy to 3 years of age in rural Senegal. J Nutr 147, 940–947. 28298540 10.3945/jn.116.243246

[15] Gewa CA & Yandell N (2012) Undernutrition among Kenyan children: contribution of child, maternal and household factors. Public Health Nutr 15, 1029–1038. 22107729 10.1017/S136898001100245X

[16] Schoenbaum M, Tulchinsky TH & Abed Y (1995) Gender differences in nutritional status and feeding patterns among infants in the Gaza Strip. Am J Public Health 85, 965–969. 7604921 10.2105/ajph.85.7.965 PMC1615541

[17] Baig-Ansari N, Rahbar MH, Bhutta ZA, (2006) Child's gender and household food insecurity are associated with stunting among young Pakistani children residing in urban squatter settlements. Food Nutr Bull 27, 114–127. 16786978 10.1177/156482650602700203

[18] Wamani H, Åstrøm AN, Peterson S, (2007) Boys are more stunted than girls in Sub-Saharan Africa: a meta-analysis of 16 demographic and health surveys. BMC Pediatrics 7, 17. 17425787 10.1186/1471-2431-7-17 PMC1865375

[19] Garenne M (2003) Sex differences in health indicators among children in African DHS surveys. J Biosocial Sci 35, 601–614. 10.1017/s0021932003006047 14621255

[20] Kruitbosch T & Heijmans DMW (2015) Influence of parental education on malnutrition in infants and children aged under-five in Kampala, Uganda. Vrije Univ Amsterdam 31.

[21] Sunuwar DR, Singh DR & Pradhan PMS (2020) Prevalence and factors associated with double and triple burden of malnutrition among mothers and children in Nepal: evidence from 2016 Nepal Demographic and Health Survey. BMC Public Health 20, 405. 32223749 10.1186/s12889-020-8356-y PMC7104542

[22] Masibo PK, Humwa F & Macharia TN (2020) The double burden of overnutrition and undernutrition in mother-child dyads in Kenya: Demographic and Health Survey Data, 2014. J Nutr Sci. 9, e5. 32042413 10.1017/jns.2019.39 PMC6984123

[23] Shinsugi C, Gunasekara D, Gunawardena NK, (2019) Double burden of maternal and child malnutrition and socioeconomic status in urban Sri Lanka. PLoS ONE 14, e0224222. 31639148 10.1371/journal.pone.0224222 PMC6805006

[24] Wondafrash M, Admassu B, Bayissa ZB, (2017) Comparative study on nutritional status of under five children with employment status of mothers in Adama town, central Ethiopia. Matern Pediatr Nutr 3, 117.

[25] Fox K & Heaton TB (2012) Child nutritional status by rural/urban residence: a cross-national analysis. J Rural Health 28, 380–391. 23083084 10.1111/j.1748-0361.2012.00408.x

[26] Bharati S, Pal M, Chakrabarty S, (2011) Trends in socioeconomic and nutritional status of children younger than 6 years in India. Asia Pac J Public Health 23, 324–340. 21551133 10.1177/1010539511403455

